# SPAN@DEM (SingHealth Patient Advocacy Network @ Department of Emergency Medicine)—A Pioneer in Emergency Department-Specific Patient Advocacy: Development Study

**DOI:** 10.2196/72552

**Published:** 2025-08-27

**Authors:** Mingwei Ng, Zhenghong Liu, Mohan Pillay, Sook Mei Chang, Boon Kiat Kenneth Tan

**Affiliations:** 1 Department of Emergency Medicine, Singapore General Hospital, 1 Hospital Drive, Outram Road, Singapore, 169608, Singapore, 65 62223322; 2 Office of Patient Experience, SingHealth, Singapore, Singapore

**Keywords:** patient advocacy, emergency department, patient-centred care, emergency physician, start-up committee, patient-centered care

## Abstract

**Background:**

Launched in January 2022, the SingHealth Patient Advocacy Network at the Department of Emergency Medicine (SPAN@DEM) represents the first emergency department-specific advocacy group in Singapore. This initiative marks a significant advancement in local patient advocacy efforts because it employs a shared collaborative model to address the needs and concerns of patients within the unique context of the emergency department environment. SPAN@DEM emerged in recognition of the limitations of existing cluster-level advocacy groups, which are inadequate to address specific challenges inherent to the fast-paced, high-pressure nature of the emergency department.

**Objective:**

In this article, we describe the establishment of SPAN@DEM, discuss the challenges and considerations encountered, and reflect on lessons gleaned through this journey.

**Methods:**

A start-up committee, comprising two emergency physicians and four patient advocates, was convened to delineate the processes required to form a new patient advocacy group. Key features of SPAN@DEM include co-leadership by an emergency physician and a patient advocate, and diverse membership composition with equal representation from health care professionals and patient advocates. SPAN@DEM convenes quarterly with informal luncheons during meetings to foster open communication between advocates and health care staff. Membership is voluntary and motivated solely by altruism, and all members are required to participate in mandatory advocacy training to empower them to provide more actionable insights.

**Results:**

Since its inception, SPAN@DEM has implemented several initiatives such as *PIKACHU* (Project to Improve next-of-Kin Advice, Communications and Helpful Updates)—a suite of quality improvement measures that resulted in improved patient and next-of-kin satisfaction rates and reduced formal communication-related complaints—and Digital FAQ—an online web-based resource designed to clarify emergency department processes for patients. SPAN@DEM advocates have also contributed to the planning, design, and transition to the new Emergency Medicine Building. More importantly, SPAN@DEM has fostered a cultural shift towards patient-centered care, with the department now routinely engaging patient advocates in decisions affecting patient and next-of-kin experience.

**Conclusions:**

SPAN@DEM exemplifies the value of specialized emergency department-specific advocacy groups in advancing patient-centered emergency care. This model may serve as an exemplar for other health care institutions seeking to enhance patient advocacy efforts.

## Introduction

The evolution of patient advocacy can be traced to its origins as disease-specific cancer support groups, which primarily focused on connecting survivors and fostering mutual support among patients. Over time, this movement has expanded to encompass critical domains such as patient safety, patient empowerment, and patient-centered care [1]. Health care institutions globally have increasingly acknowledged the importance of establishing dedicated liaisons between patients and care providers, recognizing their role as vital stakeholders who can contribute patient perspectives to formulate health care policies and provide feedback on health care processes as part of broader organizational improvement strategies [2]. This concerted effort to integrate patient advocacy across all settings within the health care delivery system [3] has led to the emergence of patient advocacy groups and organizations that are dedicated to supporting and promoting patients’ rights and interests within the health care system [4].

Emergency medicine, with its fast pace and high-acuity environment, presents unique challenges for effective patient advocacy. The emergency department functions as a critical entry point for many individuals into the hospital, frequently during times of acute distress, profound vulnerability, and heightened uncertainty. Under such stress, health care professionals may encounter difficulties in delivering compassionate care and fostering collaborative relationships with patients and their families, particularly given the overcrowded and demanding nature of the emergency department setting [5]. These factors are further complicated by the usage patterns of the emergency department. Frequent attendees may not be suitable candidates to serve as patient advocates given potential biases, while infrequent users lack insight into department operations. Moreover, the demographics of emergency department patients are notably heterogeneous, encompassing diverse cultural backgrounds, health literacy levels, and clinical needs. The absence of longitudinal follow-up further limits the continuity of physician–patient interactions as the emergency department often represents a single encounter along the patient’s broader health care journey.

Within this context, the inception of the SingHealth Patient Advocacy Network at the Department of Emergency Medicine (SPAN@DEM) in January 2022 signifies a major effort in the advancement of local patient advocacy initiatives. As the first emergency department-specific patient advocacy group in Singapore, SPAN@DEM represents a novel approach of using a shared collaborative model to address patient needs and concerns within the unique and challenging environment of the emergency department. By examining the journey that SPAN@DEM took through its conceptualization, implementation, and impact, this paper seeks to offer meaningful insights into the role that specialized emergency department-specific advocacy groups can play in shaping the future landscape of emergency care delivery.

**Figure 1.**
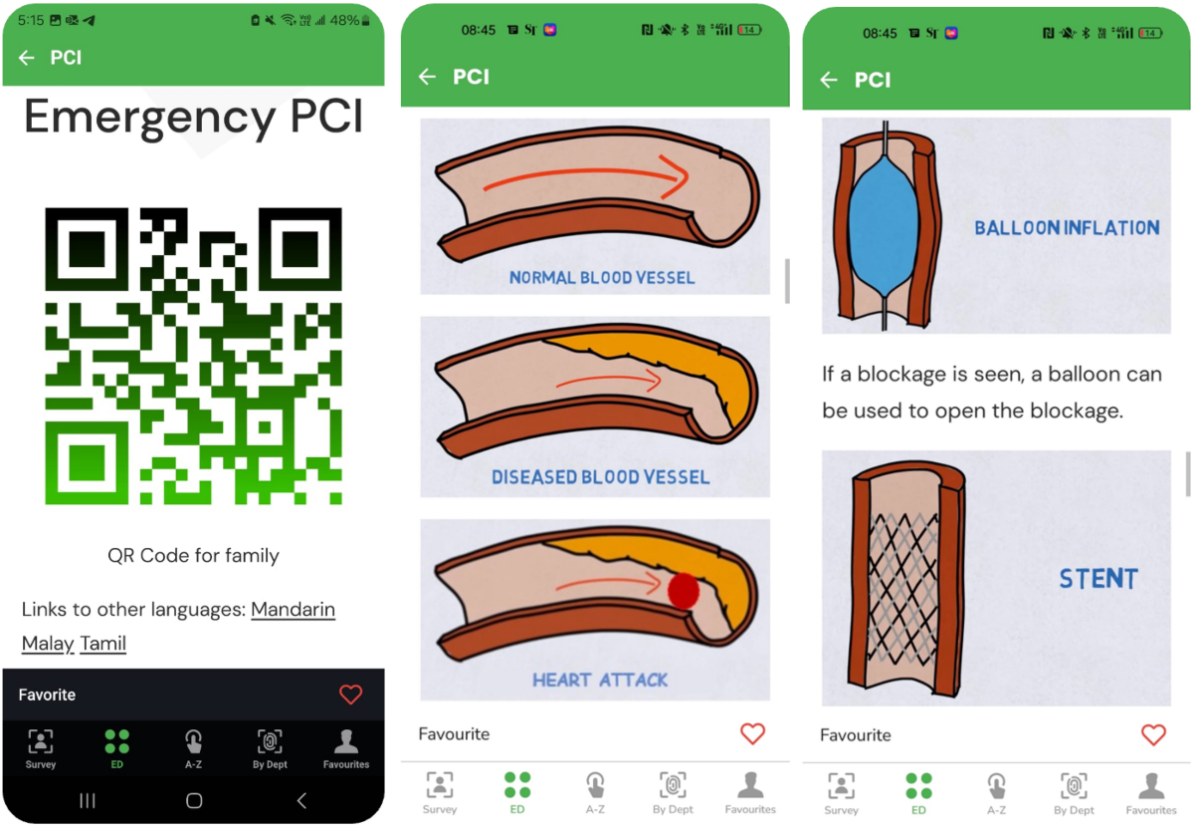
CommunicAid: a web-app to enhance patient communication. PCI: percutaneous coronary intervention.

The seeds for SPAN@DEM were sowed when a team of emergency physicians at the Singapore General Hospital (SGH) Department of Emergency Medicine (DEM) created *CommunicAid*. Emergency physicians frequently encountered difficulties in effectively communicating and obtaining informed consent for invasive procedures (such as percutaneous coronary angioplasty) from critically ill patients in high-stress, time-sensitive conditions. To address this challenge, *CommunicAid* was developed as a mobile phone application that featured standardized pictorial representations and simplified scripts to facilitate the explanation of complex medical terminology such as “stent” and “angioplasty” (Figure 1). However, the team faced challenges in selecting the appropriate communication medium and choice of terminology, which underscored the importance of incorporating the perspectives of non-medically trained laypersons to represent patient understanding more accurately. This realization resulted in a focus group discussion with patient advocates where it became apparent that the inclusion of the patient voice in department matters can be valuable. The concept of an emergency department-specific patient advocacy group was thus conceived, with *CommunicAid* serving as the impetus and foundational project for launching SPAN@DEM in January 2022.

During the development process, various models of patient advocacy were systematically evaluated, with particular attention given to successful exemplars implemented in other leading health care institutions. The emergency departments in leading hospitals like Johns Hopkins [6,7], Brigham and Women’s Faulkner Hospital [8], and UCLA Health [9] had collaborated with patients and family members to form Patient and Family Advocacy Councils. Indeed, the widespread adoption of Patient and Family Advocacy Councils internationally had prompted the Institute for Patient and Family Centered Care in the United States to create starter packs to provide structured guidance and advance such initiatives [10].

Locally, departments (like the emergency department) serve as the foundational building blocks. Several departments make up a division (like the Division of Medicine), several of which in turn make up an institution (such as SGH). These institutions collectively form a health care cluster, like SingHealth, which is responsible for overseeing one-third of the nation’s population health needs and comprises a network of four restructured public hospitals, two community hospitals, and a network of polyclinics. At the cluster level, SingHealth has embraced several patient advocacy initiatives at the macro cluster level, most notably the SingHealth Patient Advocacy Network (SPAN or SPAN@SingHealth) that launched in 2017 [3].

SPAN@SingHealth adopts a consultative model, whereby individual departments may submit requests for feedback from patient advocates on specific projects on an ad-hoc basis. Despite the positive and meaningful outcomes associated with these cluster-level initiatives, SGH DEM identified the need for a more integrated and sustained partnership with patient advocates that was grounded in a collaborative, rather than consultative, approach. SGH DEM was keen to embrace the inclusion of patient perspectives into all aspects of planning and implementation within the department, with the ultimate objective of fostering a shift towards a patient and family-centric departmental culture. In recognition of the need to bridge this mismatch between the broader, macro-level focus of cluster-level organizations like SPAN@SingHealth and the specific micro-level needs of the emergency department, SPAN@DEM was conceived as a distinct organization to better address the unique requirements of the emergency department.

As the first-ever department-level patient advocacy group locally, the principal strength of SPAN@DEM lies in its capability to deliver focused, contextually appropriate support that directly addresses the unique challenges encountered by patients in the emergency department. Operating at the department level allows SPAN@DEM to leverage intimate knowledge of emergency medicine processes and deeply integrate its advocacy efforts into routine clinical practice. In this article, we describe the process of establishing our patient advocacy group, discuss the challenges and considerations involved, and reflect on lessons learnt through this journey.

## Methods

### Ethical Considerations

As this is a descriptive paper that involves no patient recruitment or data, it did not require any ethical review.

### Start-Up Committee

A “start-up committee,” comprising two emergency physicians and four senior patient advocates from SPAN@SingHealth, was first formed to explore the processes required to create an emergency department-centric patient advocacy group. Involving SPAN@SingHealth in the initial start-up process proved valuable as it allowed the committee to avoid duplication of efforts and leverage on the parent organization’s extensive experiences and wealth of resources. This committee also engaged the SingHealth Office of Patient Experience and SingHealth Duke-National University of Singapore (NUS) Institute for Patient Safety and Quality in the discussion to provide critical insights on how to bring SPAN@DEM into fruition.

### Leadership

The start-up committee grappled with selecting an appropriate leadership model for SPAN@DEM early on. Two prevalent forms in Patient and Family Advocacy Councils were considered: one where the committee is headed solely by an advocate, and another with co-leadership shared between a health care worker and a patient advocate. While SPAN@SingHealth is headed solely by patient advocates, the decision was made to adopt the latter approach. This decision was driven by the goal of having SPAN@DEM integrate its advocacy efforts deeply within routine department practices. The dual co-leadership model leverages the unique perspectives and strengths of both health care professionals and patient advocates as the health care worker co-chair is better positioned to spearhead projects within the DEM. As most projects were initiated and implemented by the committee, it was found that proposing and following through on the projects (rather than simply consulting on projects) also gave SPAN@DEM members a strong sense of ownership and purpose in the work done.

In addition to its co-chairs, SPAN@DEM benefits from the guidance of several advisors, including the Head of Department of SGH DEM as well as representatives from the SingHealth Office of Patient Experience and the SingHealth Duke-NUS Institute for Patient Safety and Quality. These advisors are experts and are able to draw on practices implemented in other institutions to offer valuable recommendations regarding patient experience and service improvement initiatives. Securing the endorsement of senior leadership has also been critical to the success of SPAN@DEM. The initiative consistently received strong and unwavering support from the current SGH DEM Head of Department, who in his capacity as advisor to SPAN@DEM, moderated the majority of SPAN@DEM meetings alongside the Senior Nurse Manager of the department. Their regular attendance underscored the department’s strong commitment to the work and objectives of SPAN@DEM.

### Composition

To ensure diverse representation in the committee, it was decided that the committee should have a mix of specialist emergency physicians, junior doctors, emergency nurses, and department administrators. This brought the total number of 11, which was evenly split between six health care workers and five patient advocates who are patients or caregivers of patients who have previously received care from SGH DEM. Department administrative staff also served as secretariats and assisted in organizing meetings and documenting minutes. Each member was appointed for a year with an official letter and terms of reference presented at the start of each work year.

The selection process for new SPAN@DEM members was also deliberately evaluated. One approach considered was to introduce objective criteria such as frequency of emergency department visits or history of providing feedback. Members were also purposively selected to ensure sufficient representation across key demographic characteristics such as age group, gender, and underlying health conditions to better reflect the heterogeneous nature of the patient population in the emergency department. However, this presented challenges because it proved difficult to distinguish between patients with complex medical conditions requiring frequent care for legitimate clinical indications, from frequent attenders who struggle with predominantly social or mental health issues instead and contribute to disproportionate use of emergency department resources. Furthermore, the subjective and emotional nature of patient feedback resulted in much difficulty differentiating between substantive, constructive, and actionable feedback from expressions of frustration stemming from divergent worldviews that could not be realistically addressed.

The co-chairs consequently implemented a screening process and conducted interviews with prospective members to better understand their prior health care experiences and determine their suitability for membership prior to admission. This approach aimed to ensure a balanced representation of patient perspectives while maintaining the group’s focus on achievable improvements within the department’s resource constraints.

### Remuneration

Various models of remuneration were carefully considered due to the importance and sensitivity of the issue. To preserve objectivity and credibility of SPAN@DEM, it was decided that participation would be voluntary and all work would be done on an altruistic basis with no gratuities provided. Advocates would also be required to declare any potential or perceived conflict of interests (for instance, if they are affiliated to pharmaceutical organizations) and any gifts received in the course of their work for SPAN@DEM.

While advocates would not be compensated for time in monetary terms, it was decided that advocates ought to be compensated for transport and parking costs should they wish to make claims. It was also stressed in the terms of reference that advocates must respect the religious beliefs and faiths of others and strictly refrain from using SPAN@DEM as a platform to proselytize or conduct evangelism. In addition, a luncheon was organized before every meeting using department funding in recognition of the time and commitment put in by the patient advocates and health care professionals. The informal and social nature of the luncheon before each meeting (as well as the year-end parties) allowed closer friendships and bonds to be forged, which proved integral to the success of SPAN@DEM because open conversations and honest discussions can be carried out more easily.

### Meeting Frequency

The group decided on meeting quarterly. During each meeting, the committee would discuss ongoing projects as well as new ideas. While the very first meeting was conducted over video conference due to COVID-19 restrictions, subsequent meetings were conducted in-person every 3 months.

### Training

Patient advocacy involves more than just voicing personal grievances and complaints—it requires maturity for non-medical laypersons to structure and deliver feedback to health care staff in an effective manner to improve patient care. In recognition of this, SPAN@SingHealth mandated comprehensive patient advocacy training for advocates to equip them with the necessary skills to provide constructive feedback, engage in meaningful dialog, and effectively represent patient interests. It was decided that all advocates joining SPAN@DEM should undergo the same training under the Patient Advocate Communication Training program as part of the SPAN training package within the first 6 months of appointment to empower them to offer more actionable insights [2]. These workshops focus on an array of relevant topics ranging from design thinking, story-telling, to quality improvement methodology in health care and can better aid new patient advocates to work together with health care staff and achieve constructive collaboration.

### Confidentiality and Media Policy

While patient complaints and feedback that are shared during SPAN@DEM meetings are anonymized, patient advocates are allowed to do walkabouts within the department to grant them better understanding of the patient and next-of-kin (NOK) experience in the department. A non-disclosure clause was written into the terms of reference for SPAN@DEM patient advocates. This mandated that they agree to maintain the strictest confidentiality on sensitive information that they become privy to in the course of their work for SPAN@DEM. In addition, advocates are also required to inform the secretariat before engaging with media if they are approached to contribute their views in published articles or interviews in their capacity as a representative of SPAN@DEM.

## Results

Thus far, SPAN@DEM has embarked on several projects in our department. Projects were either initiated by SPAN@DEM itself, or advocates were recruited for projects initiated by the department.

### Project to Improve next-of-Kin Advice, Communications and Helpful Updates: PIKACHU

Patients and families in the emergency department often face anxiety, pain, and uncertainty, making effective communication and support critical. The rapid pace of emergency care can further limit opportunities for in-depth discussions and shared decision-making. An analysis of the complaints received by the department led to the recognition that most of these could be distilled to the lack of adequate communications with the NOK. It was in this vein that *PIKACHU* (Project to Improve next-of-Kin Advice, Communications and Helpful Updates) was started. This project sought to improve NOK satisfaction with communications within the SGH DEM and reduce communication-related complaints using systemic quality improvement methodology by introducing a bundle of quality improvement initiatives that are targeted at the root causes of patient and NOK frustration with communications (“pain points”) within the department.

Surveys were conducted to better understand the problem before several key interventions were conceptualized and implemented using a Plan-Do-Study-Act (PDSA) cycle approach [11]. Posters were put up to manage NOK expectations about likely waiting times, a patient service associate was designated as the official point-of-contact for NOK enquiries, and SMSes (short message service) were used as means to close the loop and communicate updates with NOK without compromising clinical care. *PIKACHU* demonstrated an increase in satisfaction rate among NOKs on survey scores (35% to 58%) and a significant sustained decrease in formal complaints on DEM communication matters from a monthly average of 11.3 cases pre-intervention (October to December 2022) to 0.66 cases post-intervention (July to September 2023). Nonetheless, the qualitative feedback generated from this exercise showed the team that there were still areas for improvement, especially during surge periods with lean manpower.

### Communications Workshop

Patient advocates and DEM staff analyzed actual complaints received by the department to gain insights into the nature of patient feedback. These complaints were categorized, and the most common complaints revolved around long waiting time (for consultation or for admission bed), inadequate communication (lack of updates or unclear explanation of condition or treatment instituted), and mismatched expectations. Based on these findings, the team designed four realistic scenarios to train junior doctors in managing challenging communications with the advocates portraying simulated patients to preserve authenticity.

As part of this biannual communications workshop, patient advocates also freely shared their perspectives and personal experiences with the junior doctors alongside guest speakers from the Office of Patient Experience. These light-hearted and engaging sessions aimed to foster greater empathy among the junior doctors and highlight the importance of empathy and compassionate communication amidst the stresses at work. A particularly thought-provoking observation was shared by one patient advocate: oftentimes, non-medical laypersons lack the medical knowledge to evaluate the quality of care, so they can only evaluate the quality of the communication and bedside manners and use this as a proxy in their perception of the medical care that they receive.

### Digital FAQ

Most visitors to the emergency department would be familiar with the processes in Specialist Outpatient Clinics that follow a first-come-first-served system but often feel a sense of injustice when patients who came after them are attended to first in the emergency department. In addition, unlike the relatively linear workflow of the Specialist Outpatient Clinics, a patient’s journey in the emergency department varies significantly depending on the chief complaint and complexity of the condition that the patient presents for. Some patients could get asked to have blood laboratory investigations or X-ray imaging done first or even have treatment started before consultation (“care initiated at triage”), while others may require referral to a specialist following their emergency physician consult, which results in a much lengthier wait. Other non-medical issues contribute to the overall caregiver and patient experience in the emergency department; for example, “Where can I get a meal at 2am in the morning?” or “Should I stay or should I go home?” or “Can I feed or stay with my mother?*”* are questions that the DEM staff frequently encounter.

**Figure 2.**
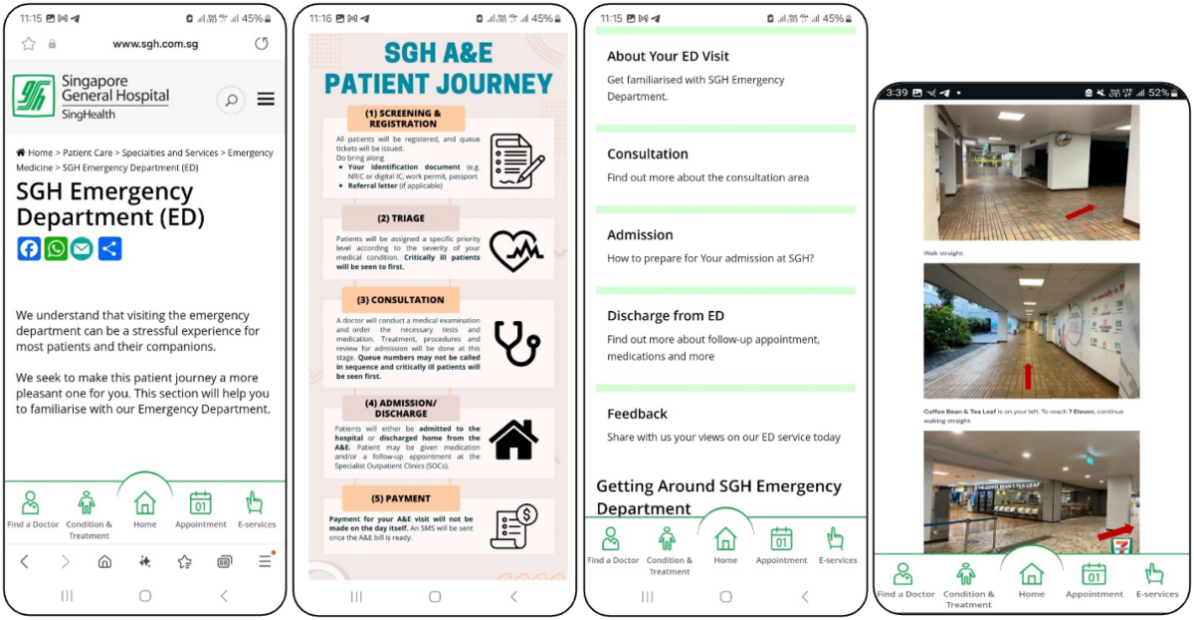
Digital FAQ resource. FAQ: frequently asked questions.

Patient advocates collaborated with the DEM staff to jointly create a series of web pages to answer these frequently encountered questions and explain the rationale behind some of the SGH DEM work processes [12].This Digital FAQ resource (Figure 2) can be accessed by patients and NOK by scanning a Quick Response code on their mobile phones and can provide patients and NOK with guidance on their journey in the emergency department.

### Department Wayfinding

**Figure 3.**
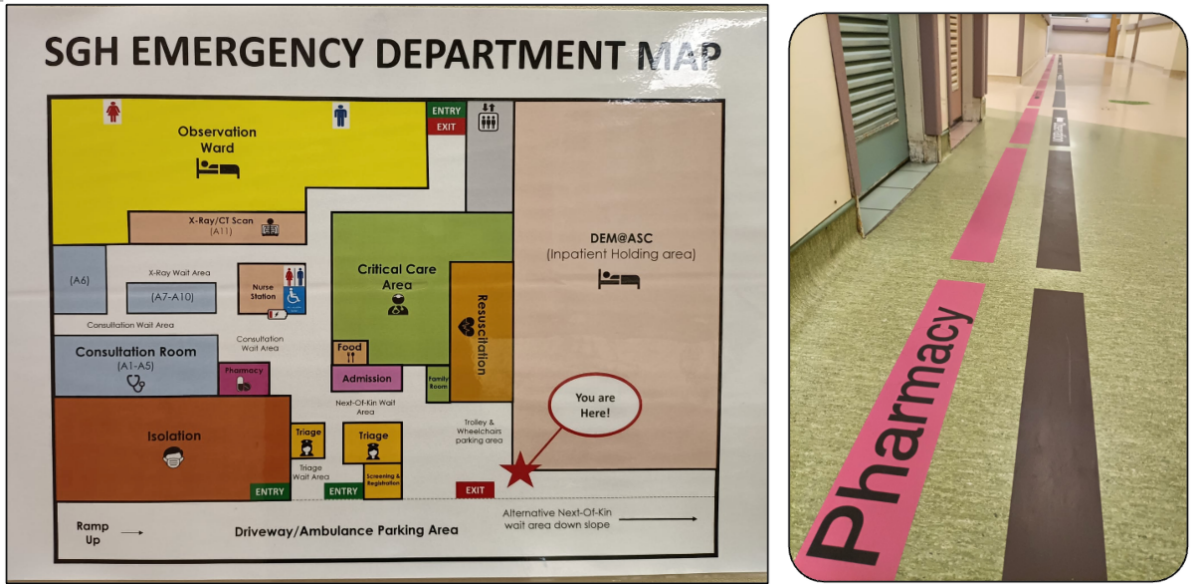
Department wayfinding.

Patient advocates from SPAN@DEM did a walkthrough of the department (with a simulated patient navigating in a wheelchair) to identify confusing signage. Several signage were amended for clarity, some were lowered to improve visualization, and loud posters were removed as they drew attention away from important signage. Directional floor stickers were also introduced (Figure 3). A new simplified map of the department layout was also created with valuable feedback from SPAN@DEM advocates (Figure 3). This did away with as many words as possible and used universal symbols to transcend language barriers and ensure that patients can understand the map more easily.

### Planning for the New Emergency Medicine Building

As SGH DEM would be moving to a new building in 2026, the patient advocates with SPAN@DEM have also been actively contributing to this endeavor by participating in various rehearsals and exercises as simulated patients, giving feedback on wayfinding and signages at the new premises. In addition, SPAN@DEM advocates have also been involved in designing and creating media material to smoothen the transition to the new building. These efforts include public education on what conditions should (or should not) be a cause to seek medical attention at the emergency department as well as how to get to the new department via various modes of transport.

### Department Culture

Beyond large-scale initiatives like *PIKACHU*, the inception of SPAN@DEM has fostered more nuanced and intangible changes within the department. SGH DEM has increasingly integrated patient advocates into routine department decision-making. During the development of the department’s sedation protocol, patient advocates highlighted their preference for the use of neutral terminology like “sedative” and “relaxant” over technical terms like “chemical restraint,” resulting in a corresponding adjustment to the wording of the protocol.

Furthermore, the close collaboration within SPAN@DEM allows the health care professionals to poll the advocates immediately and opportunistically. This facilitated rapid and prompt resolution of patient experience-related queries, such as whether patients would prefer self-registration booths at triage versus staffed counters or which patient-facing interface to display wait times was best. Emergency physicians outside of the core committee also increasingly appreciated the ability to leverage on this new-found resource and found it useful to consult the patient advocates on other matters. In some cases, SPAN@DEM advocates played “kingmaker” when the department was evenly split between several options and could not arrive at a unanimous decision. One such instance was when deciding on the naming convention within the new Emergency Medicine Building—the advocates recognized that patients and NOK may be unfamiliar with clinical jargon and proposed a zonal approach (for example, “Zone A (Critical Care)” rather than simply “Critical Care”). These developments collectively bear testimony to the subtle but significant shift in department culture towards the consistent incorporation of patient and caregiver perspectives in everyday decision-making (Table 1).

**Table 1.** Projects done by SPAN@DEM^a^ since its launch in January 2022.

Project name	Project time period	Project aim
CommunicAid	January 2022	Created pictorial diagrams to aid explanation and consent-taking of time-critical emergency department interventions
Department Wayfinding	March 2022 - June 2022	Redesigned navigational aids like maps and floor stickers to aid wayfinding within the emergency department
Patient Journey Feedback Analysis	September 2022	Conducted thematic analysis of patient feedback/complaints at various stages of the patient journey leading to a renewed focus on communication issues
Queue Viewer system	September 2022	Provided feedback to the implementation of a new queue display system in Consultation Area to improve how emergency department wait times are communicated to patients and next-of-kins
Digital FAQ	July 2022-September 2023	Created a virtual guide to address frequently asked questions for next-of-kins in the emergency department
Managing Effective Communications (I)	September 2022	Designed curriculum for communications workshop for junior doctors; participated as simulated patients to increase realism of simulation scenarios
GovTech	November 2022	Provided feedback on GovTech app-based solutions to divert cases to general practitioners as part of the GPFirst project
Surge Solutions	January-May 2023	Collectively brainstormed ideas on potential ways to alleviate stress on the ground during high patient load and emergency department lodger conditions
Managing Effective Communications (II)	March 2023	Shared patient and next-of-kin perspectives for junior doctors during communications workshops
Complex and Rare Diseases Card	September 2023 – Current	Provided feedback on creation of a medical alert card meant to improve the emergency department experience and outcomes of patients with complex and rare diseases
Managing Effective Communications (III)	October 2023	Shared patient and next-of-kin perspectives for junior doctors during communications workshops
Emergency Medicine Building	April 2024 – Current	Provided feedback about clarity of navigational aids and wayfinding signs for new emergency medicine building; filmed educational videos on which conditions should/ should not seek attendance to emergency department; actively participated as simulated patients in mock simulation rehearsals

a SPAN@DEM: the SingHealth Patient Advocacy Network at the Department of Emergency Medicine.

## Discussion

### Challenges

Assessing and demonstrating the impact of advocacy work remains challenging due to the inherently subjective nature of patient experience. Standard patient satisfaction metrics are not routinely collected in the emergency department, and developing qualitative metrics to capture less tangible benefits, such as enhanced patient dignity or emotional support, has proven complex. A mixed-methods analysis incorporating both qualitative and quantitative methodology was necessary for *PIKACHU* but was resource-intensive.

A key challenge for SPAN@DEM and similar advocacy groups face is to avoid tokenistic engagement. It is essential that health care staff refrain from engaging advocates solely for the appearance of “inclusivity” or soliciting input from advocates on matters where decisions have already been predetermined. Genuine commitment to valuing patient experiences and perspectives is essential for SPAN@DEM to be meaningful. SPAN@DEM must ensure that activities are substantive and not perfunctory; senior patient advocates have played a critical role in maintaining this standard by rigorously questioning the rationale behind proposed projects during meetings to ensure that the purpose aligned with the broader mission of SPAN@DEM.

Patient advocates, on the other hand, must adopt a broad perspective and recognize the operational constraints and limitations of the public health care system. They must understand that not all recommendations can be successfully implemented. Emotional detachment from personal experiences is important to facilitate constructive dialog. Training programs, such as the Patient Advocacy Communication Training organized by SPAN@SingHealth in collaboration with the SingHealth-Duke NUS Institute for Patient Safety and Quality, are instrumental in equipping new advocates with the skills needed to communicate their perspectives effectively and empathetically and a better appreciation of the broader public health care landscape.

Sustaining a diverse and representative membership remains an ongoing challenge that SPAN@DEM faces. Without renewal to introduce a diversity of ideas that reflect the heterogenous nature of emergency department patients, SPAN@DEM risks becoming characterized by groupthink and losing its ability to represent and remain relevant. The episodic, unscheduled, and infrequent nature of most emergency department attendances complicates membership selection, as it is inevitable that most advocates may not have recent and immediate firsthand experience of emergency department care. They thus may not fully understand the realities and frustrations faced by other patients in the emergency department. Conversely, the small group of patients with disproportionately frequent emergency department reattendances, and who therefore ironically know the department “best,” typically have a high prevalence of mental health disorders and substance or alcohol misuse and are therefore less suitable to be recruited as patient advocates.

Other more specialized departments may yield limited benefit from establishing department-specific patient advocacy groups, as existing condition-specific organizations may already have addressed these patient advocacy needs. For instance, the Department of Colorectal Surgery can work together with groups dedicated to Crohn disease, colorectal cancer, or stoma care rather than duplicate work undertaken by them. Similarly, patients managed by the Department of Neurology may already be supported by advocacy groups representing conditions such as epilepsy, Parkinson disease, or myasthenia gravis. In contrast, SPAN@DEM advocates are heterogeneous in demographic characteristics (disease type or severity, age, or socioeconomic background) and are primarily unified by their shared experiences during their emergency department visits and their desire to enhance this experience for other patients. This motivation for organizational improvement might arguably be less cohesive a bond that that of some condition-specific advocacy groups like cancer support groups, where members share common lifelong advocacy needs that extend beyond the initial emergency department encounter to inpatient care, rehabilitation, and the pursuit of resuming normalcy in life following discharge.

Despite these limitations, SPAN@DEM remains well-positioned to address the immediate and transitional needs of patients in the emergency department. The emergency department often represents patients’ first interactions with the health care system and, for many individuals, the emergency department environment remains overwhelming and stressful. SPAN@DEM plays a vital role in supporting patients and their loved ones in this regard, facilitating effective communications with the health care team and helping them navigate this critical phase of their health care journey.

### Future Directions

SPAN@DEM has served as an exemplar for others to emulate, with SPAN@DEM chairpersons having given talks and presentations at various platforms such as the Singapore Patient Advocacy Connection and the Singapore Healthcare Management Conference. SPAN@DEM co-chairs were also invited to share their experiences as a successful case study of patient advocacy to participants of the Duke-NUS Medical School Graduate Diploma in the Patient Safety and Healthcare Quality course.

As SPAN@DEM continues to evolve, the future offers exciting possibilities for enhancing patient advocacy in health care. By expanding the model to other disciplines, integrating and aligning with broader cluster-level initiatives, pursuing rigorous qualitative research and longitudinal studies on patient experience, and influencing and shaping policy, the impact of department-level patient advocacy could extend far beyond its original emergency medicine setting, potentially transforming patient experiences across the health care system.

### Conclusion

The success of SPAN@DEM underscores the value of adapting and tailoring advocacy efforts to the specific needs and challenges of individual departments. The close collaboration between advocates and clinical staff has fostered a more holistic approach to patient care in the emergency setting. SPAN@DEM marks step forward in patient-centered care within emergency medicine, potentially serving as a model for other departments and health care institutions seeking to enhance their patient advocacy efforts.

## Abbreviations

DEM: Department of Medicine
NOK: next-of-kin
NUS: National University of Singapore
PDSA: Plan-Do-Study-Act
PIKACHU: Project to Improve next-of-Kin Advice, Communications and Helpful Updates
SGH: Singapore General Hospital
SPAN@DEM: SingHealth Patient Advocacy Network at the Department of Medicine

## References

[1] Fobair P, Stearns NN, Christ G (2009). Historical threads in the development of oncology social work. J Psychosoc Oncol.

[2] Coulter A (2012). Patient engagement--what works?. J Ambul Care Manage.

[3] Sim-Devadas AL, Lakshmanan EM, Foo Z, Chang SM (2022). Building a patient advocacy network in an Asian healthcare system to enhance patient experience and patient safety. J Patient Saf Healthc Qual.

[4] Kummer HB (2024). The evolution of patient advocacy: from rights to reality. Am J Law Med.

[5] Sanocki K (2013). Patient-family centred advisory council for the emergency department [thesis]. https://idun.augsburg.edu/cgi/viewcontent.cgi?article=2445&context=etd.

[6] (2022). The johns hopkins hospital patient and family advisory councils 2022 annual report. https://www.ipfcc.org/bestpractices/annual-report-jhu-2022.pdf.

[7] Ostendarp R (2023). Improving emergency department patient experience through the engagement of a patient and family advisory council to create and implement a patient rounding tool. https://scholars.unh.edu/scholarly_projects/89.

[8] Mass general brigham and women’s faulkner hospital. ED Patient and Family Advisory Councils (ED PFAC).

[9] UCLA health. Patient and Family Advisory Councils (PFACs).

[10] Institute for patient- and family-centred care. Reports/Guidance Resources/Roadmaps.

[11] NHS england and NHS improvement. Plan, Do, Study, Act (PDSA) Cycles and the Model for Improvement.

[12] Singapore general hospital. SGH Emergency Department (ED).

